# Role of communicating diagnostic uncertainty in the safety-netting process: insights from a vignette study

**DOI:** 10.1136/bmjqs-2023-017037

**Published:** 2024-09-04

**Authors:** Caitríona Cox, Thea Hatfield, Zoë Fritz

**Affiliations:** 1 The Healthcare Improvement Studies Institute, University of Cambridge, Cambridge, UK

**Keywords:** Communication, Diagnostic errors, Patient safety

## Abstract

**Background:**

Safety-netting is intended to protect against harm from uncertainty in diagnosis/disease trajectory. Despite recommendations to communicate diagnostic uncertainty when safety-netting, this is not always done.

**Aims:**

To explore how and why doctors safety-netted in response to several clinical scenarios, within the broader context of exploring how doctors communicate diagnostic uncertainty.

**Methods:**

Doctors working in internal medical specialties (n=36) from five hospitals were given vignettes in a randomised order (all depicting different clinical scenarios involving diagnostic uncertainty). After reading each, they told an interviewer what they would tell a ‘typical patient’ in this situation. A follow-up semistructured interview explored reasons for their communication. Interviews were recorded, transcribed and coded. We examined *how* participants safety-netted using a content analysis approach, and *why* they safety-netting with thematic analysis of the semistructured follow-up interviews using thematic analysis.

**Results:**

We observed n=78 instances of safety-netting (across 108 vignette encounters). We found significant variation in how participants safety-netted. Safety-netting was common (although not universal), but clinicians differed in the detail provided about symptoms to be alert for, and the action advised. Although many viewed safety-netting as an important tool for managing diagnostic uncertainty, diagnostic uncertainty was infrequently explicitly discussed; most advised patients to return if symptoms worsened or new ‘red flag’ symptoms developed, but they rarely linked this directly to the possibility of diagnostic error. Some participants expressed concerns that communicating diagnostic uncertainty when safety-netting may cause anxiety for patients or could drive inappropriate reattendance/over-investigation.

**Conclusions:**

Participants safety-netted variously, even when presented with identical clinical information. Although safety-netting was seen as important in avoiding diagnostic error, concerns about worrying patients may have limited discussion about diagnostic uncertainty. Research is needed to determine whether communicating diagnostic uncertainty makes safety-netting more effective at preventing harm associated with diagnostic error, and whether it causes significant patient anxiety.

**WHAT IS ALREADY KNOWN ON THIS TOPIC**

- Safety-netting is a well-established strategy to mitigate against diagnostic error, but it is not clear how or why doctors communicate diagnostic uncertainty when safety-netting.

**WHAT THIS STUDY ADDS**

- Focusing on secondary care in England, this study indicates that diagnostic uncertainty is infrequently explicitly shared with patients as part of safety-netting, despite recommendations to do so.
- Exploration of doctors’ reasons behind safety-netting behaviours highlights that although many view safety-netting as an important strategy in managing diagnostic uncertainty, their communication is often tempered by concerns about worrying patients or driving inappropriate reattendance/investigation.

**HOW THIS STUDY MIGHT AFFECT RESEARCH, PRACTICE OR POLICY**

- This study highlights that, despite recommendations, safety-netting rarely involves explicit communication of diagnostic uncertainty.
- More patient-focused research is needed to better understand whether communicating diagnostic uncertainty when safety-netting changes patient health-seeking behaviours or attitudes; this could guide evidence-based best practice recommendations.

## Introduction

 Diagnostic error—missed, delayed or incorrect diagnosis—occurs frequently^1^,^3^: from <5% in ‘perceptual’ specialties (eg, radiology) to up to 10%–15% in other specialties.^4^ In specialties seeing undifferentiated patients (primary care or emergency medicine), some diagnostic error is inevitable. Not all errors reflect failure in the diagnostic process: some diseases may present atypically, or be undetectable in the early stages.^5^

‘Safety-netting’ is one strategy for mitigating against harms associated with diagnostic error.^6^,^8^ Edwards *et al* defined safety-netting as: ‘Information shared with a patient or carer, designed to help them identify the need to seek further medical help if their condition fails to improve’.^9^ Safety-netting helps to manage uncertainty in diagnosis and/or prognosis, protecting against harms arising if the initial diagnosis or predicted illness trajectory proves incorrect: it aims to ‘handle the ‘what ifs’ arising from patient reported symptoms that could, potentially, indicate a serious illness’.^10^ Safety-netting may also reduce unnecessary reattendance and the associated costs.^11^

Although safety-netting is well established, consensus on what constitutes good practice is elusive. Guidelines exist for some specific conditions (eg, NICE 2012 Meningococcal Quality Standard),^12^ but these are uncommon. Although many articles provide overlapping recommendations,^9 13 14^ there are no widely accepted guidelines on the optimal format, content or delivery of safety-netting.^1014^,^16^ Recommendations are rarely empirically grounded. A systematic review of safety-netting in primary care found that suggestions for what constitutes good safety-netting are seldom evidence based: “[t]he most compelling finding… is the lack of empirical research on safety netting.”^17^ Moreover, research on safety-netting has focused in primary care,^17^,^19^ with far less evidence on its use in secondary/tertiary care settings.^1420^,^23^

Many elements have been proposed as necessary for effective safety-netting, including *communicating diagnostic uncertainty*.^9 13 14 17 19 21 24^ A 2022 review recommended including discussion of any uncertainty in the initial diagnosis when safety-netting,^19^ while a study which used a modified Delphi process recommended: ‘If the working diagnosis is uncertain, explain the uncertainty to the patient together with the reasons for tests, investigations, watchful waiting, or a trial of management’.^25^ Various rationales have been given for recommendations to communicate uncertainty when safety-netting: not doing so may result in patients failing to appropriately return^13^ and acknowledging uncertainty may empower patients to better navigate complex diagnostic processes and minimise complaints.^9^ Despite recommendations, research suggests that diagnostic uncertainty is *not* always shared during safety-netting.^10 26^

This analysis draws from a study examining how and why hospital-based internal medicine physicians communicated diagnostic uncertainty in response to various written clinical vignettes.^27^ We identified safety-netting as an important theme. With this secondary analysis, we explore safety-netting in response to the vignettes, particularly examining the role of communicating diagnostic uncertainty as part of this process. We examine both *how* and *why* doctors safety-netted, with a focus on why doctors choose to (not) communicate diagnostic uncertainty when safety-netting. This secondary analysis does not provide an exhaustive overview of safety-netting in secondary care, but rather examines the role of diagnostic uncertainty communication as part of the safety-netting process.

## Methods

### Overview of the main study

**Figure 1:**
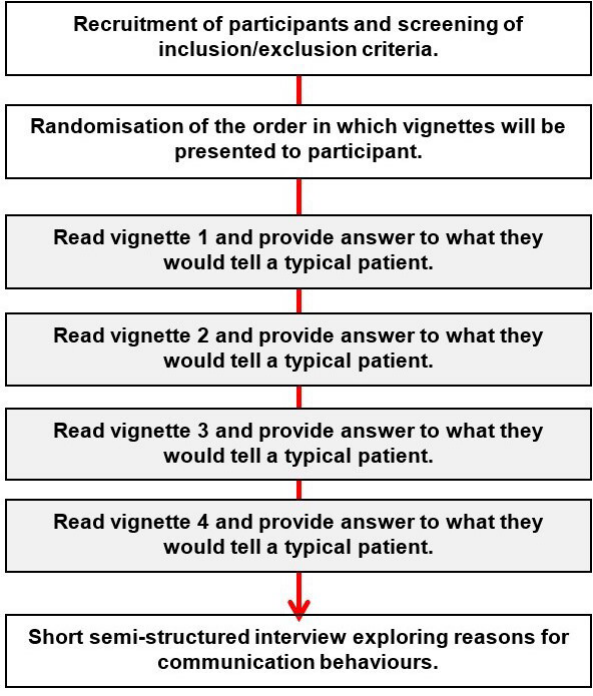
Overview of study design.

The methods of the main study are detailed elsewhere^27^ and are illustrated in figure 1. To summarise, four written vignettes involving diagnostic uncertainty were developed (using pilot-testing and expert input). All vignettes reflected cases commonly seen by medical doctors in UK acute secondary care settings (acute medical units, same day emergency care or emergency departments).

Participants (doctors who had worked in general internal medicine for (at least) three of the last twelve months) were recruited via invitations sent to National Health Service trust emailing lists at five participating hospitals. Participants were provided with four vignettes to read in a randomised order; after reading each, they were asked to tell the online interviewer what they would tell a ‘typical patient’ in the scenario. After responding to all the vignettes, a semistructured interview explored their reasons for communicating as they did; questions focused on understanding why doctors did or did not convey diagnostic uncertainty. Participants were finally asked whether they commonly see patients like those presented in the vignettes in their own clinical practice and if they found the scenarios realistic. Interviews were audio recorded and transcribed verbatim.

See table 1 for a summary of the vignettes; full copies in online supplemental materials (Although there were four vignettes in the main study, for this analysis we focused on just three (headache, change of bowel habit (CoBH) and chest pain). The fourth vignette described an anaemic patient with back pain who was being seen before further investigations were organised; safety-netting would not be typical at this stage, so we excluded the vignette from this analysis).

**Table 1:** Summary of the vignettes

Summary of vignette	Key elements of diagnostic uncertainty
40-year-old man with 3 years of intermittent abdominal pain, bloating and diarrhoea. No ‘red flag’ symptoms for cancer. A medical history of migraines and depression. No recent travel and no relevant family history. Normal examination, negative FIT (looked for blood in stool) and negative stool cultures. All tests, including FBC, LFT, thyroid function tests, coeliac serology, faecal elastase and faecal calprotectin, are normal. You believe IBS is the most likely cause of his symptoms.	The exact diagnosis here is not 100% certain—although IBS is the most likely diagnosis, there is no definitive test to confirm this. Without colonoscopy±biopsies, there is still a (very) small chance that this could be IBD or even a colorectal malignancy. Most doctors would agree that the chance of these alternate diagnoses are so low that the risks of doing further more invasive tests (such as a colonoscopy) outweigh the benefits.
45-year-old man with no medical history, who presents with central chest pain which came on with mild exertion and lasted 30 min. Normally he cycles 10 miles per day and has never had chest pain before. His maternal uncle died of a myocardial infarction aged 70, but he has no other cardiac risk factors. His examination is normal. CXR, ECG, D-dimer and serial troponins are all normal. You plan to discharge him with no further follow-up.	The investigations are all very reassuring and have essentially excluded serious pathology such as a pneumothorax, pulmonary embolus or myocardial infarction. The cause for the chest pain is not clear—it may be something benign such as acid reflux or a muscular strain, but this is uncertain. There is a small chance that this is a first presentation of angina, although this is less likely given the patient’s lack of risk factors and the fact that he cycles regularly and has never had such pain before.
30-year-old man with no medical history who presented to A&E with a severe headache, which came on at rest over a period of approximately 10 min. No associated loss of consciousness, neck pain, rash, photophobia or vomiting. His examination and observations are normal, as are his routine blood tests. He has a CT of the head within 3 hours of headache onset, which is reported by a neuroradiologist as normal. His headache has improved with paracetamol and is now a dull 3/10 severity. You are going to discharge him without a lumbar puncture (LP).	The normal examination/observations, blood tests and CT scan have essentially ruled out meningitis or a lesion inside the brain (such as a brain tumour). An important diagnosis to consider is a subarachnoid haemorrhage (SAH). Traditionally, a CT was not considered sensitive enough to rule out such a bleed, so if there was a sufficient degree of suspicion patients would go on to have an LP (which is more sensitive at detecting a small bleed). NICE guidance recommends that if the CT scan is done within 6 hours of headache onset, it can be used to exclude an SAH. For this patient, then, we cannot rule out an SAH with 100% certainty, but the risks of doing an LP most likely outweigh the benefits. We do not have a clear cause for the headache—it may be a migraine, but this is uncertain.

A&EAccident and Emergency departmentFBCfull blood countFITfaecal immunochemical testIBSirritable bowel syndromeLFTliver function testsNICENational Institute for Health and Care Excellence

### Secondary analysis on safety-netting

**Figure 2:**
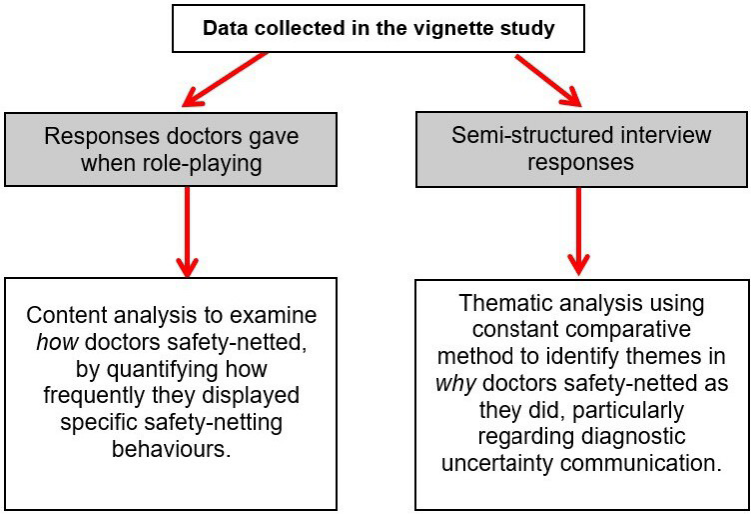
Analysis approach used in the present safety-netting study.

In the main study,^27^ we identified safety-netting as an important theme. Here, we performed a secondary analysis with particular focus on how and why participants safety-netted; figure 2 outlines our analysis approach.

To analyse *how* participants safety-netted, we examined the responses from the doctors when they were role-playing. We used a content analysis approach,^28 29^ using both deductive and inductive coding. Initial coding categories describing specific safety-netting behaviours were developed a priori (based on pilot interviews, review of relevant literature^13 18 26 30 31^ and the authors’ clinical experience). These codes were then refined after analysis of the first three interview transcripts using an inductive coding approach. We applied these finalised coding categories to the rest of the data. We quantified how frequently doctors safety-netted and how often they explicitly discussed diagnostic uncertainty when safety-netting. Quantitative results (descriptive statistics outlining the frequency of safety-netting behaviours) should be interpreted with caution given the small sample size, but we report these numbers to give readers a sense of how common certain safety-netting behaviours were in our sample.

To examine *why* participants safety-netted as they did, we analysed the semistructured follow-up interviews using the constant comparative method.^32^ Using an iterative, open coding approach, we developed codes to capture the main themes interpreted from the data; we compared data from different participants to highlight similarities and differences, considering possible reasons for these. CC and TH initially coded the first three transcripts independently, and then the whole research team met to discuss and refine the codes. CC and TH then coded the rest of the transcripts using these codes; to ensure coding consistency, they regularly independently coded the same transcript and discussed discrepancies. Higher level themes were identified and compared with existing literature to highlight trends and gaps.

## Results

Data were collected from February to March 2022. We reached theoretical saturation^33^—when we judged that further data collection was unlikely to yield new theoretical insights—after 36 interviews and closed the study.

Participants were drawn from various hospitals (from large teaching hospitals to smaller district general hospitals) and grades (from first-year qualified doctors up to consultants) (table 2). All participants felt that the scenarios presented in the vignettes were realistic and reflected their own clinical practice.

**Table 2:** Participant details

Participant characteristic	N (%)
Grade	
Consultant	10 (27.8)
Registrar or equivalent	10 (27.8)
Core trainee or equivalent	9 (25.0)
Foundation doctor	7 (19.4)
Other	0 (0)
Sex	
Female	18 (50)
Male	18 (50)
Prefer not to say	0 (0)
Ethnicity	
White	19 (52.8)
Mixed/multiple ethnic groups	2 (5.6)
Asian/Asian British	15 (41.7)
Black/African/Caribbean	0 (0)
Arab	0 (0)
Other	0 (0)
Prefer not to say	0 (0)
NHS Trust	
Hospital A (teaching hospital)	13 (36.1)
Hospital B (district general hospital)	10 (27.8)
Hospital C (district general hospital)	8 (22.2)
Hospital D (teaching hospital)	3 (8.3)
Hospital E (teaching hospital)	2 (5.6)

NHSNational Health Service

### How did participants safety-net?

We identified 78 instances of safety-netting in total, across 108 vignette encounters (table 3). Safety-netting was not universal. For instance, in the CoBH vignette, 52.8% of participants did not safety-net (no discussion about how/when patients should seek further medical advice). Safety-netting was more common in the chest pain and headache vignettes (88.9% and 80.6% respectively). Across all the vignettes, safety-netting only infrequently involved explicit acknowledgement of the possibility of diagnostic error (table 4).

**Table 3:** Content analysis of all observed instances of safety-netting, n=78

Content	Codes	Episode frequency, n (%)
Generic versus specific advice	Generic (eg, ‘any issues’, ‘any worries’ or ‘you feel unwell’)	9 (11.5)
	Specific (names specific symptom or clinical feature to be alert for)	54 (69.2)
	Both (gives specific symptoms AND also generic comment)	14 (17.9)
Number of symptoms/clinical features mentioned	1	18 (23)
	2	20 (25.6)
	3	17 (21.8)
	4	7 (9.0)
	5	6 (7.7)
	>5	1 (1.3)
Action advised	Specifically advises who to contact (eg, come back to hospital vs see your GP)	60 (76.9)
	Non-specific (eg, just advises seeking medical attention)	18 (23.1)
Strength of the endorsement	Stronger (eg, includes terms such as ‘should’, ‘must’, ‘need to’)	37 (47.4)
	Neutral (does not use stronger or weaker qualifiers)	33 (42.3)
	Weaker (eg, includes terms such as ‘can, ‘could’ or ‘feel free to’)	8 (10.3)
Timescale	Mentions a fixed time period (eg, ‘if not improving in the next day’)	1 (1.3)
	Immediate/straight away (eg, ‘come back straight away’ or ‘call 999 urgently’)	9 (11.5)
	Not specified	67 (85.9)
Focus of action	Clinician (eg, ‘*we* would then want to arrange more tests)	8 (10.3)
	Patient (eg, ‘*you* should come back then’)	51 (65.4)
	Both (gives patient action AND mentions what clinician would do)	19 (24.3)

GPGeneral Practioner

**Table 4:** Number of participants who safety-netted in each vignette

Code	N	%
Change in bowel habit vignette		
Safety nets without explicit acknowledgement of risk of diagnostic error	16/36	44.4
Safety nets with explicit acknowledgement of risk of diagnostic error	1/36	2.8
Total participants who safety-netted	17/36	47.2
Total participants who did not safety-net	19/36	52.8
Chest pain vignette		
Safety nets without explicit discussion about the possibility this is still cardiac or diagnostic error	20/36	55.6
Safety nets with explicit explanation that this could still be cardiac or diagnostic error	12/36	33.3
Total participants who safety-netted	32/36	88.9
Total participants who did not safety-net	4/36	11.1
Headache vignette		
Safety nets without explicit discussion about the possibility that this is an SAH	28/36	77.8
Safety nets with explicit explanation that this could be SAH	1/36	2.8
Total participants who safety-netted	29/36	80.6
Total participants who did not safety-net	7/36	19.4

SAHsubarachnoid haemorrhage

There was considerable variation in safety-netting content between participants, although we did not find any clear differences in how participants safety-netted based on demographic factors such as ethnicity, gender or level of seniority. Below, we present areas where we identified variation between participants, particularly considering how explicitly they acknowledged diagnostic uncertainty (if at all) as part of the safety-netting process.

#### Details provided about the symptoms to be alert for and what actions to take

A minority of participants offered generic advice (simply advising the patient to return if they were worried/concerned); more commonly, participants gave specific clinical features for patients to act on. Participants often described the most important symptom to be alert for, although the level of detail provided varied greatly. For example, in the chest pain vignette some participants simply told the patient to reattend if chest pain returned:

[I]f you get the pain again then you’re more than welcome to come back to hospital – 203

More commonly, participants provided some qualifying details (eg, being alert about the length of time the pain lasts or relevant associated symptoms).

[I]f you have severe chest pain lasting for more than ten minutes, with sweating and pain going to your arms… you should call an ambulance. – 309

A few participants gave much more detailed and specific advice, listing a range of situations when patients should return:

If you do develop any further chest pain that’s lasting longer than five to ten minutes, if it’s worrying you and certainly if there’s any associated radiation of that chest pain to the neck, down the arms, sweating, breathlessness… then the action that you took today of calling 999 and coming straight to hospital is absolutely appropriate, and I would encourage you to do that again in the future. – 202

The variation in the level of detail provided was also seen in the CoBH and headache vignettes. Participants often explained the ‘red flag’ symptoms to look out for, but the exact clinical features and level of detail varied significantly: some participants only mentioned a few symptoms to be aware of, while others listed several. In the headache vignette, most participants mentioned additional symptoms to be vigilant about (eg, visual changes, weakness, photophobia, neck stiffness or vomiting), while some only discussed what to do if the headache itself persisted or worsened.

There was also variation in what action was advised and how strongly. In the chest pain vignette, some suggested follow-up with GP, while others instructed the patient to call an ambulance.

pop to your GP if you are concerned – 304

If [the chest pains] don’t subside you should really be phoning an ambulance – 101

This variation was most marked in the chest pain vignette but was also noted in the headache scenario: some participants advised returning to ED and others suggested GP follow-up. Participants differed in how strongly they endorsed the action, with roughly equal numbers across the vignettes using neutral or strong language, and a minority using weaker language (characterised by phrases such as “you could*…*” or “feel free to*…*”).

The vast majority of participants safety-netted without specifying timelines. For example, in the headache vignette most participants gave advice focused on what to do if new symptoms developed or if the headache did not resolve, without discussing how long they expected the headache to last for:

if the headache is not settling… we can look into that further – 207

if your headache doesn’t get any better… then you need to come urgently to A&E. – 209

Only one participant gave a specific timeline for when the patient should seek further help if the headache persisted.

I would expect [the headache] to settle down and to go away entirely within the next day or two. If it hasn’t then I think it’s worth checking with the GP – 202

In the chest pain and CoBH vignettes, participants tended to focus on telling patients what symptoms to be alert for, as opposed to discussing the expected natural history of their symptoms or timeline for reconsultation.

Almost all the safety-netting we observed was passive rather than active: it involved open-ended advice about when the patient should seek further attention, rather than proactively arranging a follow-up appointment. Only one participant (in the CoBH vignette) communicated that they would arrange a review appointment when safety-netting. This was likely due to the acute secondary care context—it would often not be expected for clinicians in such settings to actively arrange follow-up with themselves when safety-netting. Relatedly, the majority of advice given was patient-focused. Participants framed their safety-netting around what actions the patient should take if symptoms persisted/worsened:

If you were to develop that… I would want you to seek medical advice – 212

Participants infrequently framed the safety-netting around the actions the medical team would take in the event of concerning ongoing/new symptoms. In this sense, the safety-netting was generally focused on equipping patients with the knowledge to act appropriately and seek medical assistance themselves.

#### Acknowledgement of diagnostic uncertainty

Participants differed in the extent to which they explicitly acknowledged diagnostic uncertainty when safety-netting. Most participants gave general advice about the need to return, without explicitly linking this to diagnostic uncertainty: they would instruct the patient to return if symptoms worsened, without acknowledging the risk of error in the working diagnosis.

[I]f you get symptoms like this again or you get a worsening headache… please do come back. – 201

[I]f the pain doesn’t get any better, or if you notice any new symptoms, like blood in your stool… do let us know. – 209

Rarely, participants acknowledged uncertainty more explicitly when safety-netting, for example, stating the working diagnosis was uncertain:

So, whilst we think this is unlikely to be heart pain, we can’t say categorically and we don’t want you putting symptoms down to indigestion in the future. – 302

Of course… we can never be completely certain about these things, so I would like you to come back to hospital if you feel like your pain is getting worse. – 306

Others were less explicit, alluding to the possibility of diagnostic error by stating that it was important for patients to return so they could be reassessed to make sure nothing had been ‘missed’:

[T]here’s always a possibility that sometimes things slip through the net. – 214

[I]f this headache suddenly gets worse… you need to come back to us again and we need to revisit the whole situation just to make sure we haven’t missed anything. – 303

Infrequently, participants discussed the differential diagnosis when safety-netting, to indirectly highlight diagnostic uncertainty:

[T]hat recent test that didn’t show any blood in it, is all quite reassuring for things like bowel cancer… I’m just raising that, not because I think you have it, but just to be aware that if you do start developing blood in your poo that would be something for us to look into in more detail again.- 306.

Although most participants did not discuss what would happen if patients were to return, some discussed the possibility of reassessment and further investigations (alluding to possible alternative diagnoses).

[If] your symptoms get worse, then we’ll need to think about reinvestigating. And there’s certainly some other tests that we could do… they’d include us looking directly at the bowel or… a CT scan. – 305

### Why participants safety-netted as they did

In follow-up interviews, we explored why participants communicated about diagnostic uncertainty when safety-netting as they did. Considerations offered by clinicians included encouraging patient reattendance in the case of further/worsening symptoms, managing patient anxiety and learning from experiences of missed diagnoses.

Many participants viewed safety-netting as an important strategy in managing diagnostic uncertainty (even if they did not explicitly share the uncertainty with the patient).

I tried to imply [uncertainty]… at the end when I was doing the safety netting, if the pain gets worse, and if it doesn’t get better… to always come back to the GP or seek medical help. I guess I didn’t explicitly state, this might be angina, but I kind of implied it at the end, that if he does get any chest pain again and he feels unwell, he needs to seek medical help for further investigation – 209

Those who discussed diagnostic uncertainty more explicitly often did so to encourage patients to return if needed.

I think it is important to be transparent with patients and not to appear dismissive so I do like to explain to them that sometimes, you know, we get things wrong or we can’t pick things up in the first instance, so… we’re here to listen and they can come back with it. – 101

I certainly don’t want to close the door in that they never think about their heart again… and then they come back ten years later because they’ve had an [myocardial infarction] and they’ve just ignored it. I want to open the door, reassure, you know, this is a dynamic thing and this is what to look out for – 312

One participant linked his acknowledgement of uncertainty to previous personal experience of diagnostic error. This experience strengthened his belief in the importance of sharing uncertainty when safety-netting, to encourage patients to feel comfortable reattending:

We all have those patients where things didn’t go well and some of them live with you and you carry those into your thinking… So there’s always that patient that lurks at the back of your brain in those situations. I always like to keep an open door to the patient because I think it’s a good strategy that you haven’t discharged them, dismissed them, said to them there’s nothing wrong here… You want to be inclusive and reassuring at the same time. – 205

Some participants felt it was important to name serious but unlikely differential diagnoses (eg, cancer) to encourage appropriate reattendance. This implicit acknowledgement of diagnostic uncertainty—through naming differential diagnoses—was often performed to make sure patients understood *why* they should reattend if symptoms persisted or worsened.

[N]othing is 100 per cent accurate when it comes to medicine… So I think it’s very important to say to the patients about the most serious conditions that you could have missed… so that in case we did miss something and the disease starts progressing… then they can come in and get it checked again. So I think safety-netting is very, very important because of diagnostic uncertainty. – 315

[B]y introducing the subject of cancer and trying to signpost those red flags I would hope that then if things do shift and do change, it’s there… [I]t’s often about giving the patient the opportunity to come back. – 202

Many discussed balancing effective safety-netting with avoiding patient anxiety. For example, in the CoBH vignette, several participants explained that they safety-netted without mentioning the possibility of cancer to avoid worrying the patient. Patient anxiety was framed as potentially harmful to both the patient and the healthcare system (it could fuel unnecessary repeat presentations/investigations).

I wouldn't really want to cause excess worry at that point by saying it could be all of these different things… based on some experience of people recurrently coming back and repeatedly having normal investigations… you want to avoid causing worry… I would definitely safety-net but I don't think there’s reason to plant that seed in their head. – 310

Yeah, I think it’s like a risk/benefit thing, isn’t it?… we still provided that safety net… but not introduced something that really is probably not the case at all but would create a lot of anxiety and worry for them. – 312

In contrast, a few participants explicitly named cancer as a possible diagnosis to encourage patient vigilance with follow-up; one participant highlighted they were more likely to do this for patients they perceived to be less likely to follow safety-netting advice.

I know it’s a bit cheeky, but… if I am worried about them, and I know they’re not going to follow up, I might try to convince them by saying… it’s very important for you [reattend], because it’s something serious that we want to rule out in your situation – 208

## Discussion

This paper explores *how* safety-netting was performed in response to diagnostic uncertainty and *why* doctors chose to (not) discuss diagnostic uncertainty when safety-netting. It builds on our previously published paper from this dataset, which investigated diagnostic uncertainty communication more generally.^27^

We demonstrated variation in the content of safety-netting. Notably, participants infrequently explicitly acknowledged diagnostic uncertainty when safety-netting: some alluded to uncertainty with vague statements (such as not wanting to ‘miss anything’), but very few clearly stated that the working diagnosis could be incorrect. Most advised patients to return if symptoms worsened or new ‘red flag’ symptoms developed, but they infrequently directly linked this to the possibility of diagnostic error. Despite this, many participants viewed safety-netting as an important tool for managing diagnostic uncertainty. Concerns about causing anxiety or driving unnecessary investigation tempered some participants’ communication.

Although there is some evidence that tolerance of uncertainty^34^ and safety-netting^23^ increases with doctor seniority, in our data there were no convincing differences between doctors of different grades in how frequently they safety-netted or in the manner in which they safety-netted. Considering the observed communication more generally (ie, not just looking at the safety-netting), we did note a slight trend towards the more junior doctors’ discussions involving less explicit acknowledgement of (and less thorough discussion about) diagnostic uncertainty. The reasons for this were not completely clear from our qualitative data; some consultants in our study alluded to having seen more cases of diagnostic error over the course of their careers, which may have made them more wary. We are, however, cautious about making strong claims about differences between participants based on their seniority, given our relatively small sample size and the fact that we did not initially set out to examine for differences in communication based on demographic factors. The extent to which experience might impact how and why doctors approach diagnostic uncertainty communication should be examined further in future research.

One of our key findings—that diagnostic uncertainty is *not* always shared when safety-netting—aligns with previous research. In a study of out-of-hours GPs, most safety-netting lacked diagnostic uncertainty communication,^26^ while another study using recorded GP consultations found that diagnostic uncertainty was communicated in 46.1% of problems discussed.^18^ Another study found that recommendations about sharing diagnostic uncertainty were only partially implemented by GPs; logistical constraints and doctors’ perceptions of patient preferences for ‘black-and-white’ answers limited such communication.^10^

If communicating diagnostic uncertainty is indeed a necessary element of effective safety-netting, our data—which suggest that this is infrequently explicitly done—have potentially serious implications for patient safety. We discuss our results in relation to two questions: (1) does communicating diagnostic uncertainty when safety-netting make it more effective in preventing harm from diagnostic error? and (2) does communicating diagnostic uncertainty when safety-netting have unintended negative effects (eg, patient anxiety or over-investigation)?

There is little evidence that safety-netting in general improves patient outcomes.^9 21^ A lack of patient-focused research is an issue: many studies focus on those providing safety-netting advice rather than those receiving it,^8 10 23 35 36^ but what a doctor explains is not the same as what a patient understands/retains.^37^ It is thus not clear how patients are influenced by different approaches to safety-netting. This lack of clarity has significant implications: patients with ‘low-risk but not no-risk’ symptoms might be particularly vulnerable to missed opportunities for cancer diagnosis.^8^ Studies examining delayed cancer diagnoses have found that being previously given a benign diagnosis for symptoms can contribute to delayed presentation,^38^ as patients often fail to appropriately re-evaluate worsening symptoms, putting faith in the ultimately incorrect benign working diagnosis initially offered.^39^ A study examining primary care diagnostic errors noted that patients did not always understand the provisional nature of the initial working diagnosis.^40^ These studies suggest that insufficient communication of diagnostic uncertainty may contribute to diagnostic delay. However, research specifically examining whether communicating diagnostic uncertainty when safety-netting actually makes patients more likely to appropriately reattend is lacking. Future studies would benefit from longer-term follow-up of patients to evaluate whether safety-netting advice alters outcomes.^18^

Patient fear can be a barrier to help-seeking behaviour, particularly in the context of possible cancer.^3841^,^43^ Some of our participants suggested that discussing the full differential diagnosis and acknowledging uncertainty might induce patient anxiety. These participants would often tell patients what symptoms to be alert for, without necessarily disclosing the significance of these symptoms: they equipped patients with the knowledge of *what* to look for, but not *why* to look for it. The perception that safety-netting (particularly mentioning diagnoses such as cancer) might be anxiety-inducing has been noted in primary care.^8 30 42^ In a study which used a modified Delphi approach to gather consensus opinion on safety-netting, the authors commented that “it is possible that, in the case of suspected cancer, communication of uncertainty is balanced with minimising anxiety associated with a possible cancer diagnosis,”^25^ specifically calling for further research exploring how uncertainty should be best communicated.

This perception—that detailed safety-netting may cause unnecessary anxiety—is not corroborated by patients. Black *et al* study found that patients sometimes perceive vague or passive safety-netting as dismissive, and when patients do not properly understand the GP’s diagnostic strategy it can cause frustration and worry.^30^ It is thus plausible, in contrast to doctors’ concerns, that patients may be *more* worried by vague or passive safety-netting, which does not specifically acknowledge diagnostic uncertainty. Ultimately, a lack of patient-focussed research means that the extent to which patients are worried by discussion of diagnostic uncertainty is unclear. We therefore echo Black *et al* in their assertion that “future safety netting research should measure patient understanding and reconsultation behaviour, developing strategies that improve these outcomes without raising unnecessary anxiety.”^30^

In contrast, a minority of our participants purposefully mentioned serious differentials to increase the likelihood of a patient following safety-netting advice—even if this caused worry. These participants used patient anxiety as a positive tool to encourage patients to appropriately reattend. A similar phenomenon was demonstrated by a study of cancer safety-netting, which found that GPs might deliberately increase the patient’s level of concern about their symptoms to ensure they accept responsibility for follow-up; as one GP in that study noted, “[i]t’s never nice to frighten people but I think under certain circumstances you probably have to, to a certain extent.”^44^ The concept of patient worry as a protective health factor has been explored elsewhere (eg, a study which found worry was associated with greater motivation to engage in information-seeking about advanced care planning).^45^

Safety-netting as a mechanism for transferring responsibility from doctor to patient is particularly important in acute secondary care, where the doctor–patient relationship is inherently transient. Evans *et al* discuss how responsibility for follow-up can be passed from GP to patient during safety-netting; patients holding sole responsibility are at risk if they are not provided with sufficient explanation about diagnostic uncertainty.^44^ While in primary care it may be feasible for the GP to share more of the responsibility for appropriate follow-up, when a clinician is discharging a patient from an acute secondary care setting this often represents the end of that particular doctor–patient relationship. This can account for our finding that the focus of the safety-netting action tended to be on the patient rather than the clinician. In their review of primary care safety-netting, Friedemann Smith *et al* concluded that patients are more likely to follow safety-netting advice if they understand who holds responsibility for the action, emphasising that effective safety-netting relies on understanding between GP and patient.^19^ We argue that the secondary care clinician has a particular responsibility to make sure the patient has sufficient information to take on the responsibility for reattending: there is an even greater need for safety-netting advice provided at the end of an acute secondary care encounter to include a clear explanation of diagnostic uncertainty.

A small number of our participants suggested that sharing diagnostic uncertainty when safety-netting might drive inappropriate representation or over-investigation. There is little evidence to support these concerns. A review of ‘clinically unnecessary’ patient use of emergency/urgent care identified risk minimisation as a driver, for example, caused by anxiety due to uncertainty about symptoms^46^; in this context, clear discussion about diagnostic uncertainty may be beneficial. Safety-netting has been identified as important in helping patients navigate the ‘Goldilocks Zone’ in healthcare: patients face difficulties in identifying when it is ‘just right’ to seek medical advice, and safety-netting may reduce the moral burden on them in making such decisions.^47^

### Strengths and weaknesses

Our vignette methodology facilitated controlled study of communication: by providing all participants with identical clinical information, we could examine differences between participants in their safety-netting. Our vignettes were externally valid: all participants reported them to be realistic and reflective of everyday clinical practice. Participants were recruited from a range of geographical locations and were evenly distributed across different grades (from first year qualified doctors to consultants).

There are, however, inherent limitations to using hypothetical vignettes. What participants communicated in this artificial setting may not reflect what they would communicate in a real consultation. Responses may be influenced by social desirability bias (where participants present an idealised version of their normal communication). Other factors which may alter real safety-netting communication were absent from our study, such as logistical constraints and patient cues. We did not examine non-verbal components of safety-netting and nor did we consider issues surrounding health literacy (eg, the patient’s comprehension of information provided).

A limitation is that the main study was designed to investigate the communication of diagnostic uncertainty, not just safety-netting. As such, the semistructured interviews were not focused on safety-netting per se, but rather communication of uncertainty in general (although many participants discussed safety-netting). This paper presents an analysis of a prominent theme, and should be interpreted as such: there are elements of safety-netting which we did not explore, and we do not suggest that our discussion is comprehensive.

## Conclusion

This study highlights that explicit communication of diagnostic uncertainty is uncommon as part of the safety-netting process, despite various existing recommendations which specifically advocate its disclosure. While many saw safety-netting as a key strategy in managing diagnostic uncertainty and mitigating against diagnostic error, their communication was often tempered by a desire to avoid worrying patients.

If and how discussing diagnostic uncertainty influences the effectiveness of safety-netting is unclear: research examining whether it makes patients more likely to appropriately seek medical advice if symptoms worsen is lacking. It is also unclear whether patient anxiety, potentially induced by discussing diagnostic uncertainty, should be viewed as harmful or as a helpful tool for encouraging patients to take safety-netting advice seriously. The need to provide sufficient information—including about diagnostic uncertainty—is particularly important in acute secondary care settings where the doctor–patient relationship is transient, when patients must be empowered to take on responsibility for appropriate reattendance. There is a need for future research to garner patient perspectives and to determine the impact of different methods of safety-netting on health-seeking behaviours, to build an evidence base to inform safety-netting guidelines.

## supplementary material

10.1136/bmjqs-2023-017037 online supplemental material 1

## Data Availability

Data are available on reasonable request.
