# Supplementary material for: Role of communicating diagnostic uncertainty in the safety-netting process: insights from a vignette study
Source: BMJ Qual Saf. 2024 Sep 4;33(12):e017037. doi: 10.1136/bmjqs-2023-017037 (PMC11671892; doi:10.1136/bmjqs-2023-017037)
Supplement: online supplemental material 1 [file bmjqs-33-12-s001.pdf]

Appendix: copy of written vignettes

Vignette 1

- You are looking after a 40-year-old man who has had vague abdominal symptoms on and off for 3 years: he has mild intermittent lower abdominal pain (which is relieved by defecation), bloating and change of bowel habit (intermittent episodes of diarrhoea).
- He has no weight loss, no vomiting, no blood or mucus in the stool. He does not get symptoms at night.
- He has a PMH of migraines and mild depression, for which he takes citalopram.
- He has no FHx of note.
- He has no recent travel history.
- Physical examination is normal, and observations are all normal.
- He had a recent negative FIT test (Faecal Immunochemical Test – looks for blood in stool).

All bloods, including FBC, LFT, thyroid function tests, coeliac serology, faecal elastase and faecal calprotectin, are normal. Stool culture is normal.

The patient does not voice any specific worries or concerns regarding the cause of his symptoms when you ask about this.

You believe IBS to be the most likely cause of his symptoms.

Please tell me exactly what you would tell a typical patient next.

Vignette 2

- You are looking after a 75-year-old lady with a background of longstanding rheumatoid arthritis (for which she is stable on long term methotrexate). She takes no other regular medication.
- She has been feeling more tired than usual for a few months, and also has some mild lower back pain for the last few weeks.
- She has no other symptoms; she has not lost any weight and has not had any night sweats.
- Recent bloods taken as part of methotrexate monitoring shows a mild normocytic anaemia (Hb 105). U&Es and LFTs are normal.

You feel the most likely diagnosis is anaemia of chronic disease, but you want to order some further investigations to rule out more serious conditions (in particular myeloma). As part of your work-up you plan to organise iron studies, B12 and folate, LDH, bone profile, a blood film, serum electrophoresis and serum free light-chain assay and urinary protein/electrophoresis.

The patient does not voice any specific worries or concerns regarding the cause of her symptoms when you ask about this.

Please tell me exactly what you would tell a typical patient about the next steps.

Vignette 3

- You are looking after a 45-year-old man with no PMH, who presents with central chest pain, which came on after mild exertion and lasted for approximately 30 minutes. He is normally able to exercise without trouble: he cycles 5 miles in and out of work each day sometimes quickly.
- The pain was dull, 5/10 severity, did not radiate anywhere and was not associated with any palpitations. There were no exacerbating or relieving factors. He has never had pain like this before.
- He does not have any shortness of breath or cough.
- He has no PMH. He is a non-smoker and does not drink alcohol.
- He has a FHx of IHD: maternal uncle died of an MI aged 70.
- Physical examination is normal and his observations are all in the normal range.
- An ECG shows normal sinus rhythm with normal axis and no evidence of any ischaemic changes.
- A CXR is normal.
- 2 Troponins taken 3 hours apart are normal. A d-dimer is normal.

When you ask, the patient tells you that he was concerned that it was his heart causing the pain.

You plan to discharge him with no further follow-up. Please tell me exactly what you would say to a typical patient in explaining this to him.

Vignette 4

- You are looking after a 30-year old man who presented to A&E with a severe headache. The headache came on at rest over approximately 10 minutes, and he describes it as the 'worst of his life'.
- He has had no loss of consciousness, neck pain, photophobia or vomiting.
- Physical examination is normal, with no evidence of meningism and no focal neurology.
- He had no PMH and no FHx of note.
- Routine bloods are normal. He has a CT head within 3 hours of headache onset, which is reported as normal.
- He now has a mild residual headache after being given paracetamol – 3/10 severity, dull generalised pain. He still has no other symptoms.
- You are leading the post take round and have decided that an LP is not warranted, and he can be discharged.

The patient does not voice any specific worries or concerns regarding the cause of his symptoms when you ask about this.

Please tell me exactly what you would tell a typical patient.
